# Sniffer dogs can identify lung cancer patients from breath and urine samples

**DOI:** 10.1186/s12885-021-08651-5

**Published:** 2021-08-13

**Authors:** Charlotte Feil, Frank Staib, Martin R. Berger, Thorsten Stein, Irene Schmidtmann, Andreas Forster, Carl C. Schimanski

**Affiliations:** 1 2nd Department of Internal Medicine, Municipal Hospital Darmstadt, Grafenstraße 9, 64283 Darmstadt, Germany; 2 Toxicology and Chemotherapy Unit, German Cancer Research Center, Heidelberg, Germany; 3 Institute for Medical Biostatistics, Epidemiology and Informatics, Johannes Gutenberg-University Mainz, Mainz, Germany; 4 Pulmonologist’s Office Darmstadt, Darmstadt, Germany

**Keywords:** Lung cancer, Olfactory detection, Volatile organic compounds

## Abstract

**Background:**

Lung cancer is the most common oncological cause of death in the Western world. Early diagnosis is critical for successful treatment. However, no effective screening methods exist. A promising approach could be the use of volatile organic compounds as diagnostic biomarkers. To date there are several studies, in which dogs were trained to discriminate cancer samples from controls. In this study we evaluated the abilities of specifically trained dogs to distinguish samples derived from lung cancer patients of various tumor stages from matched healthy controls.

**Methods:**

This single center, double-blind clinical trial was approved by the local ethics committee, project no FF20/2016. The dog was conditioned with urine and breath samples of 36 cancer patients and 150 controls; afterwards, further 246 patients were included: 41 lung cancer patients comprising all stages and 205 healthy controls. From each patient two breath and urine samples were collected and shock frozen. Only samples from new subjects were presented to the dog during study phase randomized, double-blinded. This resulted in a specific conditioned reaction pointing to the cancer sample.

**Results:**

Using a combination of urine and breath samples, the dog correctly predicted 40 out of 41 cancer samples, corresponding to an overall detection rate of cancer samples of 97.6% (95% CI [87.1, 99.9%]). Using urine samples only the dog achieved a detection rate of 87.8% (95% CI [73.8, 95.9%]). With breath samples, the dog correctly identified cancer in 32 of 41 samples, resulting in a detection rate of 78% (95% CI [62.4, 89.4%]).

**Conclusions:**

It is known from current literature that breath and urine samples carry VOCs pointing to cancer growth. We conclude that olfactory detection of lung cancer by specifically trained dogs is highly suggestive to be a simple and non-invasive tool to detect lung cancer. To translate this approach into practice further target compounds need to be identified.

## Background

### Lung cancer: mortality, risk, and screening methods

Lung cancer is the leading oncological cause of death in western countries and it is the second most frequent cause of death after cardiovascular diseases in Germany [1]. The WHO estimated 2.09 million newly diagnosed lung cancer patients in 2018 worldwide.

The prognosis of lung cancer is unfavorable as indicated by the death of 16.382 female and 29.692 male patients in Germany 2017 [1]. The mortality rate increases with age and reaches a maximum in the age group from 80 to 84 years [2]. An advanced age at diagnosis is considered as unfavorable prognostic factor [3]. In 2017 the median age of death was 71 years for women and 72 years for men [1].

Although early diagnosis of cancer using effective screening methods is crucial for successful treatment, no surveillance tools exist. In contrast to other cancers, there is still no early test for lung cancer [2]. Some tumor markers are available; however, due to limited sensitivity and specificity the current German S3 guideline does not recommend the routine determination of tumor markers for primary or recurrent lung cancer diagnostics, nor their use as screening tool [2].

Even if effectiveness has now been proven by some studies [3, 4], the current German S3 guideline does not yet recommend general low-dose computed tomography for screening in risk patients. Reasons among other include risks such as false positive findings with corresponding unnecessary follow-up interventions and radiation exposure.

Considering the frequency of deaths from lung cancer in Germany and worldwide the development of a screening tool for early detection of lung cancer would be of high importance, since early detection measures could lead to earlier diagnosis and thus result in better outcome.

### Sniffer dogs for cancer detection identifying volatile organic compounds (VOCs)

Dogs have a highly sensitive olfactory system, which is already used in several ways. The olfactory organ of the dog is similar to the human olfactory organ but has some peculiarities that explain the better olfactory perception. Dogs can differentiate 10.000–100.000 times better between different smells compared to humans and are able to identify volatile organic compounds (VOCs) starting from a concentration of 1:1 trillion VOCs [5]. Labrador, Retriever and the German Shepherd seem to be best suited for olfactory detection [6].

Recently, much attention has been given to the use of odors emitted in the form of VOCs as diagnostic biomarkers. In several studies, sniffer dogs were trained to discriminate cancer samples (breath, urine, cancer tissue) in different media. Across published studies, a sensitivity of 78% and a specificity of 71.5% related to olfactory detection of lung cancer samples by sniffer dogs was reported [7–11]. Those study results are encouraging but published studies differ in terms of the experimental setup, kind of odor samples, sample collection methods, dogs’ characteristics and dog training methods as well as in results presented in terms of detection sensitivity and specificity [12, 13]. In addition, it could not be clarified so far what chemical compounds dogs respond to and the quantity of these compounds. As several studies show that dogs are able to detect lung cancer either in urine or in breath yielding different results, it was an important part of this study to test if the combination of both procedures could lead to a higher detection rate. Therefore, it was the aim of this study to evaluate the capability of a classically conditioned domestic dog to accurately distinguish samples of lung cancer patients of all tumor stages in urine and breath from healthy controls without preselecting included patients with regard to smoking behavior or other lifestyle habits. By combination of urine and breath samples.

## Methods

### Study design

This was a prospective, double-blind, clinical trial including 432 patients, that were recruited at the municipal hospital Darmstadt (Klinikum Darmstadt GmbH) in cooperation with a pulmonologist’s office. The trial was approved by the ethics committee of the chamber of physicians Hessen, Germany (project no FF20/2016) and was conducted from May 2016 to August 2017. The study included a conditioning phase and a study phase. Patients were separated into 2 groups: Cancer patients were defined as patients with histologically proven lung cancer, and controls were defined as participants with no detectable tumor and a physiological heart and lung auscultation.

**Fig. 1:**
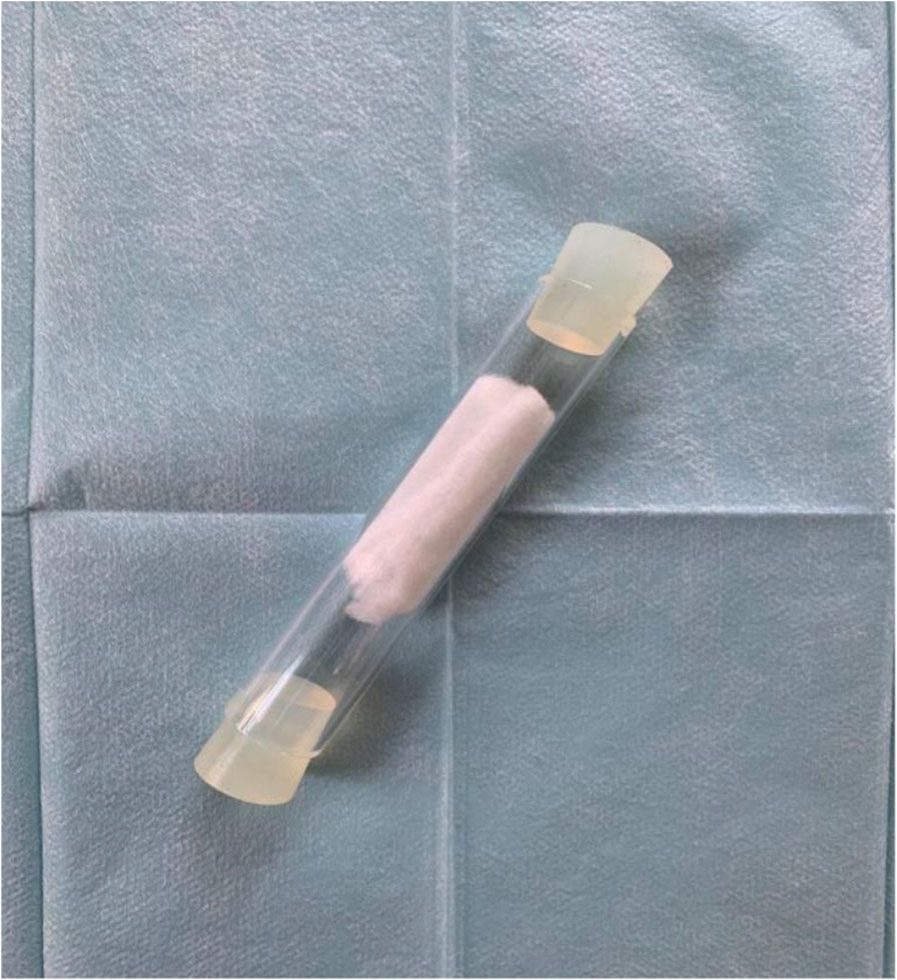
Example of a test tube (breath sample)

**Fig. 2:**
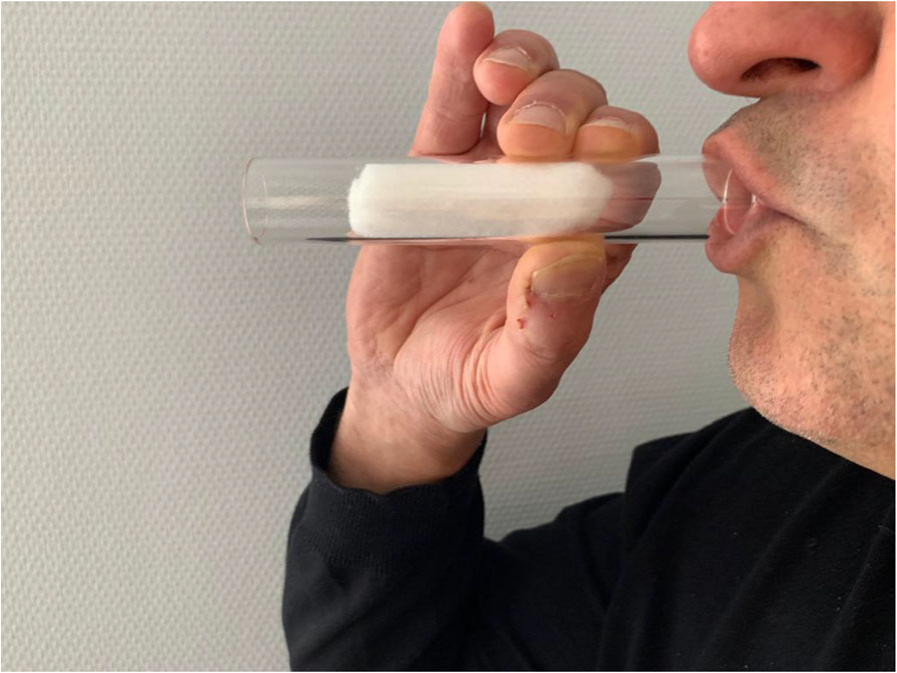
Presentation of the handling of the breath tubes during delivery of the breath samples

In the conditioning phase, a Labrador dog was trained with samples of 36 cancer patients and 150 control patients, in total 186 patients; afterwards, further 246 patients in the age range 45 to 80 years were included into the study phase: 41 patients presented with a biopsy confirmed lung cancer enclosing all different stages. In addition, 205 healthy individuals with no cancer history participated and served as control samples (Table 1). From each patient two breath samples and two urine samples (total samples = 864 samples each) were collected (see Fig. 1 and Fig. 2) and were shock frozen immediately at − 80 °C.

**Table 1:** Characteristics of both patient collectives – conditioning phase and study phase

		Conditioning Phase (*n* = 186)		Study Phase (*n* = 246)	
Collective		**Cancer**	**Control**	**Cancer**	**Control**
Total number of patients (n)		36	150	41	205
Gender (female/male)		17 / 19 ≜	74 / 76 ≜	12 / 29 ≜	85 / 120 ≜
		47.2% / 52.7%	49.3% / 50.6%	29.3% / 70.7%	41.5% / 58.5%
Age in years (mean)		66.72	62.91	65,63	61.71
Smoker / Non-smoker		17 / 19 ≜	38 / 112 ≜	22 / 19 ≜	59 / 146 ≜
		47.2% / 52.7%	25.3% / 74.7%	53.7% / 46.3%	28.8% / 71.2%
Renal infection (yes / no)		1 / 35 ≜	7 / 143 ≜	1 / 40 ≜	4 / 201 ≜
		2.8% / 97.2%	4.7% / 95.3%	2.4% / 97.6%	2.0% / 98.0%
Regular use of alcohol (yes / no)		6 / 30 ≜	15 / 135 ≜	6 / 35 ≜	28 / 177 ≜
		16.7% / 83.3%	10.0% / 90.0%	14.6% / 85.4%	13.7% / 86.3%
Tumor entities	**SCLC**	8 ≜ 22.2%		10 ≜ 24.4%	
	**Squamous epithelial cell cancer**	11 ≜ 30.6%		12 ≜ 29.3%	
	**Adenocarcinoma**	17 ≜ 47.2%		18 ≜ 43.9%	
	**Large-cell lung cancer**	0		1 ≜ 2.4%	
UICC-Stage	**Stage I**	3 ≜ 8.3%		2 ≜ 4.9%	
	**Stage II**	6 ≜ 16.7%		6 ≜ 14.6%	
	**Stage III**	13 ≜ 36.1%		11 ≜ 26.8%	
	**Stage IV**	14 ≜ 38.9%		22 ≜ 53.7%

Sample preparation and collection was highly standardized: Urine samples were self-collected from a specimen cup filled with spontaneous urine by the patient. There was no further differentiation concerning the timing of the urine sample, e.g. early morning or midstream urine in order to avoid any sample preselection. All participants were asked for signs of urinary tract infections; results of urine rapid tests were incorporated if they were already available. For breath samples all test tubes were prepared under aseptic conditions by one person from the study team wearing aseptic gloves. Furthermore, the same person assisted each patient during sample acquisition within the study, and all samples were collected in the same room of the municipal hospital Darmstadt to avoid any contaminating smell as well as to achieve constant conditions during the sample preparation and acquisition process. Test tubes were prepared according to the process described by Ehmann et al. [9]: using 120x22mm glass tubes (Gaßner, Glastechnik GmbH, München, Germany). They were filled with one hydrophobic and one hydrophilic siliconized 2.5 × 6.0 cm piece of polypropylene cotton wool (cotton wool by Asota Ges.m.b.H, Linz Austria; siliconization by CHT Bezema, CHT R. Breitlich GmbH, Tübingen, Germany). During preparation of test tubes and sample collection the working area was cleaned and disinfected using Cleanisept Wipes (Dr. Schumacher GmbH, Malsfeld, Germany), covered by Molinea Plus 40x60cm tissues (Paul Hartmann AG, Heidenheim, Germany). The study team members wore aseptic gloves, surgical caps, face mask and sterile gowns.

One urine and one breath sample of each patient was preserved for another study at the German Cancer Research Center, and the other one was used in this study for presentation to the dog. In a randomized, double-blinded manner breath and urine samples were presented separately to the dog. This resulted in a specific conditioned reaction pointing to the cancer sample. In contrast to most other studies, only samples from new subjects were presented to the dog during study phase. This was to rule out that the dog reacted to an already known odor instead of identifying the specific cancer odor.

### Eligibility criteria

Participants of both groups (cancer group and control) were 45–80 years of age and able to provide informed consent. Eligible patients for the cancer group had histologically confirmed lung cancer. Patients with completely cured tumors, other malignancies, with tumors of other than bronchial origin or with lung metastases originating from another cancer were not eligible for this study.

Patients of the control group were either hospitalized in the municipal hospital Darmstadt for other reasons, or they were hospital staff or patients from the nearby pulmonologists office in Darmstadt. Healthy volunteers were also accepted. Their samples were taken at the study’s office in the municipal hospital Darmstadt. All included patients of the control group as well as all healthy volunteers had no cancer history and a non-pathological heart and lung auscultation.

### Study evaluations

Cancer patients underwent a lung function test less than 5 days prior to bronchoscopy using a bodyplethysmograph “MasterScreen Body”, Carefusion Germany GmbH. Only forced expiratory volume (FEV1) was collected for the study with a FEV1 > 60% of the target value corresponding to a minor obstruction, a FEV1 = 40–60% of the target value corresponding to a medium obstruction and a FEV1 < 40% corresponding to a major obstruction. A FEV1 of > 80% was defined as normal. All bronchoscopies were performed using a bronchoscope of Olympus Medical Systems Corp., series EVIS EXERA III (BF-P190 or BF-1TH190) or series EVIS EXERA II (BF-1 T180). The bronchoscopy was evaluated as abnormal if the investigators were able to display direct or indirect tumor signs. At each bronchoscopy, samples of endobronchial tumor, the bronchial mucosa, or lung tissue (transbronchial biopsy) and adjacent lymph nodes were collected, and a lavage cytology was obtained.

In addition, the local lymph node situation and, if necessary, the spread of the primary tumor was evaluated using endobronchial ultrasonography (EBUS, PENTAX Europe GmbH, Ultrasound-Videobronchoscope EV-1970UK).

All tissue samples were analyzed histopathologically at the Department of Pathology at the Municipal Hospital Darmstadt and distinction was made between a small cell lung cancer (SCLC) and a non-small cell lung cancer (NSCLC).

In all patients with confirmed lung cancer a computer tomography (CT) was done at the Department of Radiology t and the tumor markers carcinoembryonic antigen (CEA), cytokeratine fragment (CYFRA) 21–1 and neuronspecific enolase (NSE) were determined at the central laboratory of the Municipal Hospital Darmstadt.

### Sniffer dog, its conditioning process and study setup

Samples were evaluated by a 7-year old Golden Retriever dog trained for one year by a professional dog trainer of TEAMCANIN, Filderstadt, Germany. The training of the dog during the conditioning phase as well as sample detection during the study phase took place at the dog training facilities of TEAMCANIN. Otherwise the dog was living at his home place together with its owner. This dog was already trained to discriminate different smells e.g. chamomile tea, cinnamon or coffee.

For this study, the dog was conditioned by a classical conditioning method, called the clicker method: a correct indication of a sample was initially rewarded by food along with a specific click from a clicker device. During the conditioning phase the dog was trained to accept the click sound as the only positive reinforcement sign. Duration of the conditioning phase was about one year with training once or twice a week. Week days and training hours changed randomly. During training time the dog was able to move freely, thus he was not leashed. First, only cancer samples were presented to the dog, afterwards more and more control samples were presented together with the cancer sample until the setup goal of the study phase was achieved (one cancer sample and five control samples).

Samples were presented in an aluminum tray and each sample was covered with a funnel that was discarded after each presentation to ensure that the dog’s nose was not affected by the sample’s smell. The dog was standing during sample presentation, sniffing the samples with its nose and indicating a cancer sample by pausing more than five seconds with its nose in the funnel of a sample.

Samples involved in the conditioning phase were kept in a standard freezer at − 20 °C until they were presented to the dog.

Urine and breath samples were always presented separately.

The position of the cancer sample was chosen by dice and the sample was positioned in the aluminum tray as indicated by the dice number. This process was blinded to the dog and its trainer. Dog and owner entered the room with the samples to be studied only after the assistant had left. When the dog identified a sample as cancer sample as it was taught, the trainer reported the number of the sample to his assistant waiting in the room next to the study room. The assistant replied whether the dog’s identification was correct, and the dog was then rewarded or not. The dog was rather quick sniffing the samples and indicating a cancer sample – one round of sample detection lasted about 10 s.

The study phase took place during May 2017 and August 2017.

### Study phase

Each urine and breath sample was evaluated only once and was successfully presented to the dog in a randomized, double-blinded manner. In total 41 cancer samples and 205 control samples were evaluated per sample type. Only samples that had not been used during the conditioning phase were evaluated during the study phase to make sure that the dog did not recognize the smell of an already known sample.

### Statistics

Results were recorded in an Excel 2010 table and stored at the 2nd Medical Department II of the Municipal Hospital Darmstadt.

For the main outcome the detection rate was computed as the proportion of correctly identified cancer samples, this was compared to 78% using one-sided exact binomial tests, since 78% was the mean values for sensitivity to the literature [9–11, 14, 15] and the proportion of correctly identified cancer samples is approximately equal to the sensitivity. In addition, 95% Pearson Clopper confidence intervals were provided.

To assess statistical significance in subgroup comparisons, Pearson Chi-square test and the Fisher exact test were used. Results were considered statistically significant if the *p*-value was ≤0.05.

A correction for multiple testing was not done as the intent of the analysis was exploratory. Missing values were excluded from the statistical analysis.

While the design of the study did not allow to compute sensitivity and specificity of the sniffer dog, a formula was derived which related underlying sensitivity and specificity to the probability of correctly identifying the cancer sample among six samples presented to the dog. Thus, pairs of values for sensitivity and specificity could be obtained that were compatible with the observed detection rates. Further, lower bounds for sensitivity and specificity, given the observed detection rates, could be computed.

With s = sensitivity, r = 1 – specificity and ^+^ the probability of correctly detecting the cancer sample, it holds that $s=r\frac{6{\pi}^{+}-{\left(1-r\right)}^5}{1-{\left(1-r\right)}^5}$. The derivation of the formula and details of the calculations are provided as supplementary material. Possible pairs of values of values for sensitivity and specificity are displayed graphically.

## Results

### Patient collectives

Conditioning phase: Urine and breath samples of 186 patients were collected from which 36 patients were assigned to the cancer group and 150 to the control group. The mean age was 63.7 years in the control group and 66.7 years in the cancer group. Slightly more men than women participated (50.6% vs 49.3 and 52.7% vs 47.2%, respectively) and the gender distribution was almost equal in both groups.

Study phase: Urine and breath samples of 246 patients were collected who had not participated before in the conditioning phase. Out of those, 41 patients were included in the cancer group and 205 patients in the control group. The mean age was 62.4 years (control) and 65.6 years (cancer group), respectively. The proportion of males and females in the cancer and control group did not differ significantly (*p* = 0.145): the cancer group consisted of 29 males (70.7%) and 12 females (29.3%), and the control group of 120 males (58.5%) and 85 females (41.5%). Signs for urinary tract infections had been found in about 2% in the cancer and control group. Characteristics of both groups are shown in Table 1.

**Fig. 3:**
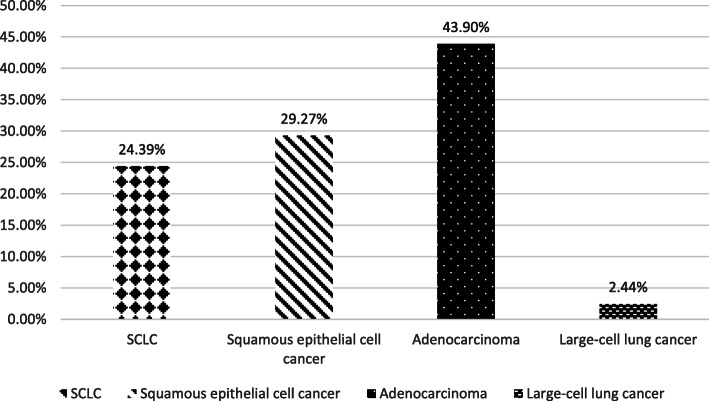
Tumor entities study phase

The tumor entities of the study phase were distributed as shown in Fig. 3.

Most of the lung cancer diseases were diagnosed as UICC stage 4 (53.7%), followed by UICC stage 3 (26.8%). Only 4.9% of the patients were in UICC stage 1 and 14.6% were in UICC stage 2. The distribution of UICC stages was similar in both, the study phase and the conditioning phase.

### Bronchoscopy, CT, lung function tests, tumor marker

Bronchoscopy revealed endobronchial pathological findings in 23 of 41 cancer patients resulting in a sensitivity of 56.1%. The lowest sensitivity was achieved in the diagnosis of adenocarcinomas (33.3%). It was not possible to determine the specificity of the method since no bronchoscopy was done in patients of the control group.

By CT examination 100% of carcinomas were diagnosed correctly; again, the specificity of this method could not be determined, as control group patients did not receive CT diagnostics.

Data of the lung function test (FEV1) were available in 35 patients. Mean FEV1 was 62.8% ± 23.0% corresponding to a mild obstruction. In 9 out of 35 patients, there was no pathological finding.

**Fig. 4:**
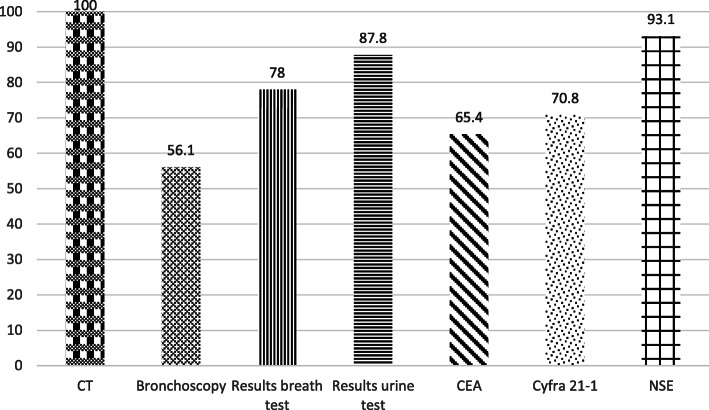
Comparison of the sensitivity of the diagnostic procedures in relation to detection rates in lung cancer using breath tests

Results of tumor markers (CYFRA 21-1, CEA, NSE) were available in 30 patients. For 29 out of these 30 patients at least one tumor marker was positive. NSE was the tumor marker showing the highest sensitivity with 93.1%; specificity of the method was not determined. In Fig. 4 comparison of the sensitivity of the diagnostic procedures compared to the detection rates using breath samples (approximately equal to sensitivity) is shown.

### Results of breath tests – detection rates

The dog correctly predicted cancer in 32 of 41 breath samples, corresponding to a detection rate of 78% (95% CI [62.4, 89.4%]). This did not differ significantly from the reference value of 78% (*p* = 0.5851). Results are depicted in Table 2.

**Table 2:** Evaluation of breath samples: Detection rates

Results breath samples			
	**Sample chosen**		**Total**
	**cancer**	**control**	
**Number of sample groups**	32	9	41
**Percentage**	78.0%	22.0%	100.0%

Looking at tumor entities the highest detection rate (90.0%) was found for SCLC. Large-cell lung cancers were not evaluated in detail since there was only one patient present with this diagnosis. Detection rate of adenocarcinomas was 72.2 and 74.2% for the total group of NSCLC. However, differences between histological subtypes are not statistically significant (*p* = 0.710). Results are summarized in Table 3.

**Table 3:** Evaluation of breath samples: Detection rates by tumor entity

	Tumor entity		Results breath test		Total
			false	correct	
	SCLC	Number of samples	1	9	10
		Percentages	10.0%	90.0%	100.0%
	Squamous epithelial cell cancer	Number of samples	3	9	12
		Percentages	25.0%	75.0%	100.0%
	Adenocarcinoma	Number of samples	5	13	18
		Percentages	27.8%	72.2%	100.0%
	Large-cell lung cancer	Number of samples	0	1	1
		Percentages	0.0%	100.0%	100.0%

SCLC: small cell lung cancer

Considering the Union for International Cancer Control (UICC) stages the detection rate of the breath test was not evaluable at UICC stage 1 due to the low case number. Detection rate improved with higher UICC stages and was 66.7% at UICC stage 2, 72.7% at UICC stage 3, and 81.8% at UICC stage 4, respectively. The differences in detection rates between stages were not statistically significant (*p* = 0.793).

The detection rate in cancer patients with a normal FEV1 was 88.9%. In patients with severe obstruction, it was 83.3%. The differences in detection rates of the breath test by FEV1 were not statistically significant with *p* = 0.646.

Correlating test results with bronchoscopy findings, there was no statistically significant difference in the detection rates of the breath test (*p* = 0.970).

**Fig. 5:**
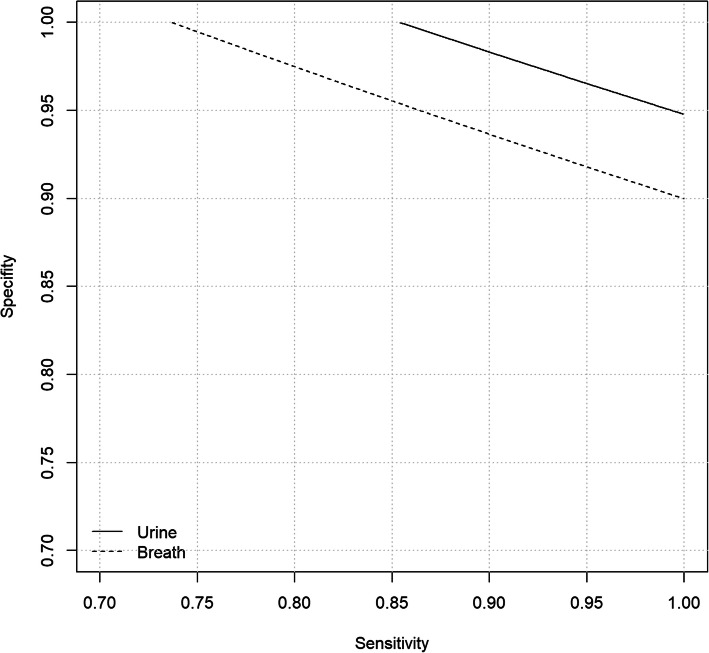
Combinations of sensitivity and specificity compatible with observed detection rates

The lower bound for sensitivity (at specificity = 100%) was derived as 73.7%. Similarly, specificity was at least 90% (at sensitivity = 99.9%). For all possible pairs of values for sensitivity and specificity see Fig. 5.

### Test in urine – detection rate

The dog correctly predicted cancer in 36 of 41 samples, resulting in a detection rate of 87.8% (95% CI [73.8%, 95.9. The detection rate did not differ significantly from the reference value of 78% (*p* = 0.0865). Results are depicted in Table 4.

**Table 4:** Evaluation of urine samples – detection rate

Results urine samples			
	**Sample chosen**		**Total**
	**cancer**	**cancer**	
**Number of sample groups**	36	5	205
**Percentage**	87.8%	12.2%	100.0%

Evaluating the detection rate of the test in relation to tumor entities the highest detection rate (91.7%) was found for squamous epithelial cell cancer. Again, due to the low case number, large-cell lung cancers were not taken into consideration. Detection rate for the detection of SCLC was 90.0% and it was 83.3% for adenocarcinomas. However, differences between histological subtypes are not statistically significant (*p* = 0.865). Results are summarized in Table 5.

**Table 5:** Evaluation of urine samples – detection rate by tumor entity

	Tumor entity		Results urine test		Total
			false	correct	
	SCLC	Number of samples	1	9	10
		Percentages	10.0%	90.0%	100.0%
	Squamous epithelial cell cancer	Number of samples	1	11	12
		Percentages	8.3%	91.7%	100.0%
	Adenocarcinoma	Number of samples	3	15	18
		Percentages	16.7%	83.3%	100.0%
	Large-cell lung cancer	Number of samples	0	1	1
		Percentages	0.0%	100.0%	100.0%

SCLC: Small cell lung cancer

Looking at UICC stages the highest detection rate in detection of cancer samples could be demonstrated for samples of patients with UICC stage 3 (100.0%) followed by samples of patients with UICC stage 4 (86.4%). However, results were not statistically significant.

Correlating test results with bronchoscopy findings, there was no statistically significant difference in the detection rate of the urine test.

The lower bound for sensitivity (at specificity = 100%) was derived as 85.4%. Similarly, specificity was at least 94.8% (at sensitivity = 99.9%). For all possible pairs of values for sensitivity and specificity see Fig. 5.

### Comparison of the results of the test in breath and in urine by tumor entity and UICC stage

Combining both techniques, the dog correctly identified 40 of 41 cancer samples, leading to an overall detection rate of 97.6% (95% CI [87.1, 99.9%]) which was significantly higher than the reference value of 78% (*p* = 0.0005). SCLCs were detected with equal frequency in both breath and urine samples. Samples of adenocarcinoma as well as of squamous epithelial cell cancer were detected slightly more frequently in urine samples but this difference was not statistically significant. UICC stage 2–4 cancer was more frequently found by the dog in urine samples than in breath samples.

## Discussion

It was the purpose of this study to evaluate the capability of a classically conditioned domestic dog to accurately discriminate urine and breath samples from lung cancer patients of all tumor stages from healthy controls in a strongly standardized and controlled setting of a prospective trial.

### Study population

The study population as defined by the in- and exclusion criteria reflected the population in which the development of cancer is more likely and would therefore benefit most from lung cancer screening. Based on the data of the German S3-guideline for lung cancer, males are more frequently affected by lung cancer than females [2], hence 149 male and 97 female patients participated in this study. According to the Robert Koch Institute, the mean age of disease onset was 66 years in males and 64 years in females in 2016 [1]. The incidence increases starting at the age of 35–39 years and peaks at the age of 80–84 years. The mean age of the study population was 65.6 years corresponding to the mean age of patients published by the Robert Koch Institute, however, an extension of the inclusion age up to and including 85 years would have defined the affected age group more precisely.

Some authors describe that the amount of dissolved substrates or VOCs in exhaled air depends on the lung volume [16]. Therefore, FEV1 values of cancer patients were determined. We could not confirm that the ability of dogs to correctly detect cancer samples was compromised when evaluating samples from patients with a limited FEV1. In this study, the detection rate in breath samples from patients with normal FEV1 was only slightly better than that of breath samples from patients with severe obstruction (88.9% vs. 83.3%). However, this statement is impaired by the low number of patients with a medium or severe obstruction. Mass spectroscopic analyses are warranted to get further insights into the influence of a low flow perhaps resulting in a lower concentration of VOCs.

### Sniffer dogs can distinguish VOCs from cancer patients and set up during the study

The race of the conditioned dog as well as factors like age, diet, motivation or health status could influence the suitability of a dog. To get more objective results it might be useful to have the same samples evaluated by several dogs, as it was already tried in other studies [9, 13, 17, 18]. Another factor influencing detection results had been demonstrated by Biehl et al., who described different outcomes depending on previous experiences of the dog, e.g. police dog vs. household dog vs. tumor sniffing dog with a better outcome for experienced dogs. However, due to the low number of dogs included in detection dog studies, it is difficult to prove an influence of the individual dog on the result statistically [13, 17].

Dogs have a pronounced olfactory memory. Therefore, repeatedly presented patient samples during the conditioning and/or the study phase may result in a dog recognizing an already known sample and thus being incorrectly conditioned to the detection of already presented samples rather than to the olfactory detection of cancer-specific VOCs. In order to exclude this possibility, all presented patient samples had been presented only once during the study phase. This procedure reduced bias to a minimum due to residence or food within the same patient. Control of any lifestyle factor was not intended, as the aim of this study was to develop a screening method for lung cancer that could be used everywhere and for everyone, independent from the lifestyle. While the development of an electronic nose is the ultimate goal of our study, the sniffer dog’s identification of lung cancer, as presented here, was only the first step towards the electronic nose and will be followed by identification of a VOD signature within the same samples that had been analyzed here. However, we employed highly stringent criteria to avoid any bias due to smells derived from preparation of test tubes, sample preparation and acquisition. The carefully controlled preparation of our test tubes is described in the Material and Methods section; the same study group member preparing the test tubes for breath samples was also assisting patients during sample acquisition. Furthermore, all samples were taken in the same room of the municipal hospital Darmstadt. Similarly, during the engagement of the sniffer dog, actions were taken to rule out any cross contamination of samples or smells. For example, the setting for the sniffer dog was always prepared by the same person, which was different from the person accompanying the dog; funnels applied to the test tubes facilitating the dog sniffing at each sample were used only once; position of positive samples among the negative samples was randomly assigned and blinded to the person leading the dog.

In the present study, encouraging results of the olfactory detection of lung cancer from urine and breath samples of patients could be shown. Experimental studies using trained dogs to identify breath odor markers of human cancer have been analyzed and compared with the authors’ own results. The mean sensitivity reported in all previously published studies dealing with the olfactory detection of lung cancer was 78%, the mean specificity was 71.5% [9–11, 14, 15].

Our results concerning sensitivity for the urine samples with a lower bound of 85.4% were better than the reference value of 78%. Breath samples alone might be inferior regarding sensitivity – but only if specificity is above 98%. Specificity was substantially better than the reference value of 71.5%, both for breath and urine samples. Combining breath and urine samples is likely to lead to higher sensitivity as in 40 of 41 cases the cancer sample was detected, this will come at the cost some decrease in specificity, however, it is unlikely that the combined specificity will be below 85%.

A limitation of the design is that was impossible to obtain precise estimates for sensitivity and specificity. In order to obtain not only bounds but precise values for sensitivity and specificity, some kind of replication of the sniffing experiment with the same dog would be necessary.

It could be shown that the dog was generally better in detecting lung cancer from urine and breath samples than bronchoscopy. In this study, a sensitivity of only 56.1% could be achieved by bronchoscopy vs a sensitivity of at least 84.5% in urine and of at least 73.7% in breath samples by olfactory detection. With a sensitivity of 100%, CT is the diagnostic gold standard but due to the associated radiation exposure, its use should be limited to a population at risk. The use is also limited by the high rate of false positive results [19]. Here, the combined analysis of urine and breath samples yielding an identification of 40 out of 41 samples corresponding to a detections rate of 97.6% is a promising approach, which might fill this diagnostic gap.

Thus, the olfactory detection of lung cancer is a promising alternative: The collection of breath and/or urine samples is non-invasive and there is no risk for the patient. Therefore, a precise specification of a risk population is not necessary, since even with a sensitivity and specificity < 100% there would be a benefit for the patient. Due to the simplicity of the sample collection, this test would be feasible at many locations; it is less expensive than a CT examination, requiring a relevant radiation exposure and a contrast medium with their associated risks and can be repeated at any time. Another major advantage of sniffer dogs is that they could start patient screening right after their training without further delay for patients at risk, while the development of an electronic nose requires a yet to determine profound understanding of the VOCs indicating lung cancer with comparable sensitivity and specificity.

Disadvantages and limitations of this method - general aspects: Lung cancer is among the most common cancer entities, therefore a high number of sufficiently trained dogs would be required, which seems to be unrealistic. Training of dogs is very individual and time consuming and needs to be performed by a number of professional dog trainers taking race, age, previous experience, etc. into account. Furthermore, the sniffer dogs would have to maintain their high level of training over a long time period and - even in case this could be feasible - their performance still depends on their daily status and motivation as living individuals. Considering these aspects only, this seems to be hardly feasible in clinical practices of family doctors. In addition, factors that could disturb the olfactory detection by sniffer dogs needs to be identified.

There is still another point, which had already been discussed in the literature and is of importance: It could be assumed that, apart from the smell of smoke or the change in VOCs, e.g. due to a urinary tract infection, other circumstances such as drugs or hospital odor could influence the dogs’ accuracy [12]. Finally, variables like room temperature, climate conditions or humidity might also affect the sensitivity and specificity of sniffer dogs during the patient screening process.

Since it is not known which VOCs or combination of VOCs indicates cancer, it is hardly possible to determine processes in the body or environmental factors, which might have an influence on the expression or detection of these VOCs.

A study on chronic obstructive pulmonary disease (COPD) patients identified drugs as possibly disturbing factors but the inflammation itself was excluded [9]. In addition, the specific scent of hospitals was identified as potentially disturbing in other studies [12, 20].

In a study by McCulloch et al. household dogs were trained to accurately distinguish breath samples of lung and breast cancer patients from those of controls. A correlation between current tobacco consumption and the sensitivity of the olfactory detection could be demonstrated [7]. In contrast, in a study of Ehmann et al. [9] lung cancer was identified with an overall sensitivity of 71% and a specificity of 93% but the detection was independent from the presence of tobacco smoke and food odors. However, if tobacco consumption should result in a poorer sensitivity and specificity of olfactory detection of lung cancer, this method would be not suitable for screening.

In conclusion, lung cancer screening by sniffer dogs seems to be affected by too many variables to become highly reliable. Therefore, the aim is to identify electronically the VOCs that indicate cancer.

### Application of an electronic nose in cancer detection

Using an electronic nose for diagnosing cancer had been used for a variety of different cancers with lung cancer being the most intensively studied. Bladini C et al. provided an extensive overview on the variety of cancers studied as well as the current technically available systems discussing their advantages and disadvantages [21]. The authors identified 37 articles dealing with commercial and non-commercial electronic nose systems published until January 2020. These studies used various statistical approaches, e.g., principle component analysis, support vector machine and logistic regression analysis, to identify “the lung cancer breathprint” being able to distinguish between healthy controls and lung cancer in different stages. Among these Li et al. [22] used an array of 14 different sensors in combination with an in depth data pre-processing and, among others, support vector machine processing for classification, reaching a sensitivity of 91.6% and a specificity of 91.7%. The authors concluded that these studies displayed satisfying results with different technologies, which could be used in clinical practice with desirable technological development [21].

In contrast, identification of a breathprint of lung cancer patients using currently available electronic nose systems was the approach presented in this study: Identification of VOC biomarkers by analysis of exhaled breath in lung cancer and non-cancer samples.

Although 24 VOCs have been identified to date as potential lung cancer markers, e.g. aldehydes, 2-butanone and 1-butanol [23], there is little consensus among studies dealing with identification of lung cancer-specific VOCs. In addition, only few studies link VOC levels in patients’ breath with approaches that employ sniffer dogs, as we will do following this study. This approach is to serve as positive reinforcement.

For a successful olfactory detection of lung cancer it is crucial to understand which compounds are tumor-specific, in order to use such VOCs as true tumor markers. As already mentioned, previous studies comparing the dog’s detection rates of synthetic samples vs cancer samples achieved varying results [24–26]. Lung cancer cells seem to produce VOCs, which differ from normal cells, or a larger amount of VOCs compared to normal cells. Sponring et al. demonstrated that certain compounds like 2-ethyl-1-hexanol and 2-methylpentane can be cancer cell derived and thus may be indicative of the presence of a tumor [27]. Another difference between normal cells and cancer cells is the increased metabolism of substances like several types of aldehydes and butyl acetate by cancer cells resulting in a lower concentration of those VOCs [27]. Currently available literature suggests that a combination of several VOCs may indicate the presence of cancer better than a single cancer-specific VOC, [12]. Buszewski et al. [25] stated, that the signature odor of cancer that dogs use for differentiation between samples may be related to specific qualitative or quantitative olfactory impressions produced by a mixture of VOCs.

The identification of cancer-specific VOCs will therefore be a challenging objective of further studies in order to develop a non-canine screening method for lung cancer that provides an identical or higher sensitivity and specificity as achieved by the canine nose. Among these studies Li W et al., outlined a research protocol for construction of a model for early prediction of lung cancer based on exhaled biomarkers using gas chromatography-mass spectrometry [22]. As follow-up analysis of the study presented here, we will use the second sample of each study participant to identify potential target molecules for cancer detection by mass spectrometry similar to the outline published by Li W et al. [22].

## Conclusions

It is known from current literature that breath and urine samples carry VOCs pointing to cancer growth. We conclude that olfactory detection of lung cancer by specifically trained dogs is highly suggestive to be a simple and non-invasive tool to detect lung cancer. To translate this approach into practice further target compounds need to be identified.

## Data Availability

All data generated or analyzed during this study are included in this published article.

## Abbreviations

CEA: carcinoembryonal antigen
COPD: chronic obstructive pulmonary disease
CT: computer tomography
CYFRA: cytokeratine fragment
EBUS: endobronchial ultrasonography
EBUS-TBNA: EBUS controlled transbronchial needle aspiration
FEV: forced expiratory volume
NSCLC: non-small cell lung cancer
NSE: neuronspecific enolase
SCLC: small cell lung cancer
UICC: Union internationale centre le cancer
VOC: volatile organic compound
